# The Ecology of Antibiotic Use in the ICU: Homogeneous Prescribing of Cefepime but Not Tazocin Selects for Antibiotic Resistant Infection

**DOI:** 10.1371/journal.pone.0038719

**Published:** 2012-06-25

**Authors:** Andrew N. Ginn, Agnieszka M. Wiklendt, Heather F. Gidding, Narelle George, James S. O’Driscoll, Sally R. Partridge, Brian I. O’Toole, Rita A. Perri, Joan Faoagali, John E. Gallagher, Jeffrey Lipman, Jonathan R. Iredell

**Affiliations:** 1 Centre for Infectious Diseases and Microbiology, Westmead Hospital, Sydney, New South Wales, Australia; 2 Centre for Research Excellence in Critical Infection and Sydney Institute for Emerging Infections and Biosecurity, University of Sydney, Sydney, New South Wales, Australia; 3 Westmead Millennium Institute, Westmead, New South Wales, Australia; 4 National Centre in HIV Epidemiology and Clinical Research, University of New South Wales, Sydney, New South Wales, Australia; 5 Queensland Pathology, Royal Brisbane Hospital, Brisbane, Queensland, Australia; 6 Intensive Care Unit, Westmead Hospital, Sydney, New South Wales, Australia; 7 Ludwig Engel Centre for Respiratory Research, Westmead Hospital, Sydney, New South Wales, Australia; 8 Department of Microbiology, Princess Alexandra Hospital, Brisbane, Queensland, Australia; 9 Intensive Care Unit, Royal Brisbane and Women’s Hospital, Brisbane, The University of Queensland, Brisbane, Queensland, Australia; Charité-University Medicine Berlin, Germany

## Abstract

**Background:**

Antibiotic homogeneity is thought to drive resistance but *in vivo* data are lacking. In this study, we determined the impact of antibiotic homogeneity per se, and of cefepime versus antipseudomonal penicillin/β-lactamase inhibitor combinations (APP-β), on the likelihood of infection or colonisation with antibiotic resistant bacteria and/or two commonly resistant nosocomial pathogens (methicillin-resistant *Staphylococcus aureus* and *Pseudomonas aeruginosa*). A secondary question was whether antibiotic cycling was associated with adverse outcomes including mortality, length of stay, and antibiotic resistance.

**Methods:**

We evaluated clinical and microbiological outcomes in two similar metropolitan ICUs, which both alternated cefepime with APP-β in four-month cycles. All microbiological isolates and commensal samples were analysed for the presence of antibiotic-resistant bacteria including MRSA and *P. aeruginosa*.

**Results:**

Length of stay, mortality and overall antibiotic resistance were unchanged after sixteen months. However, increased colonisation and infection by antibiotic-resistant bacteria were observed in cefepime cycles, returning to baseline in APP-β cycles. Cefepime was the strongest risk factor for acquisition of antibiotic-resistant infection.

**Conclusions:**

Ecological effects of different β-lactam antibiotics may be more important than specific activity against the causative agents or the effect of antibiotic homogeneity in selection for antibiotic resistance. This has important implications for antibiotic policy.

## Introduction

Immediate effective antibiotic therapy is one of the most important intervention in severe sepsis, but ultimate survival is determined before cultures become positive and antibiotic susceptibilities available [1], [2]. Broad-spectrum antibiotics such as carbapenems (e.g. meropenem), antipseudomonal penicillin/β-lactamase inhibitor combinations (APP-β) like piperacillin/tazobactam (TZP) and ticarcillin/clavulanate (TIM), and broad-spectrum cephalosporins such as cefepime are commonly used, but are restricted in many countries, with the aim of minimising development of antibiotic resistance.

The clinical benefit of antibiotic-intensive strategies such as selective decontamination of the digestive tract [3] comes at the cost of increased antibiotic resistance in the microflora [4]. Conversely, antibiotic restriction may reduce antibiotic resistance. In the “antibiotic cycling” strategy, potent antibiotics are alternated in order to allow specific resistance to subside. Initially promising reports [5] have been followed by numerous studies with mixed results. A study comparing 4-month cycles of carbapenems, third-generation cephalosporins and APP-β suggested a relationship between highly homogeneous antibiotic selection pressure and resistant infection [6], while another using carbapenems, quinolones, cephalosporins and APP-β found no increase in resistance in the microflora during or after the cycling period [7]. Expert reviewers conclude that more data are needed, as many questions remain unanswered [8], [9], but mathematical models of antibiotic cycling predict that homogeneous selection pressure will increase antibiotic resistance [10], [11].

Our primary aim was to determine whether cycles of relatively homogeneous β-lactam use, alternating cefepime and APP-β as the core of empiric antibiotic therapy for sepsis in ICU, resulted in increased antibiotic resistance. We defined this as the acquisition at any site of either MRSA or *P. aeruginosa* (two commonly resistant nosocomial pathogens) or of any bacteria resistant to cefepime, APP-β, or gentamicin, and considered these separately. A secondary aim was to determine whether the institution of antibiotic cycling in ICU was associated with adverse clinical outcomes, specifically relating to mortality and length of stay, in light of previous findings of increase mortality associated with empiric use of cefepime in the critically ill [12].

## Methods

### Study Design and Population

We varied the β-lactam component (cefepime vs APP-β) of empiric antibiotic prescribing in four month cycles in two well-matched high-acuity metropolitan Australian ICUs. No attempt was made to influence prescribing for known or suspected aetiologies, but those judged in need of empiric broad-spectrum β-lactam therapy by the caring physician received cycle-specified β-lactam (cefepime or APP-β) with other antibiotics as deemed appropriate. Standard infection control procedures applied, according to matching guidelines in both institutions. Approval to conduct the study, under a waiver of consent, was obtained from the relevant Human Research Ethics Committees of the Sydney West Area Health Service and the Royal Brisbane and Women’s Hospital.

**Figure 1:**
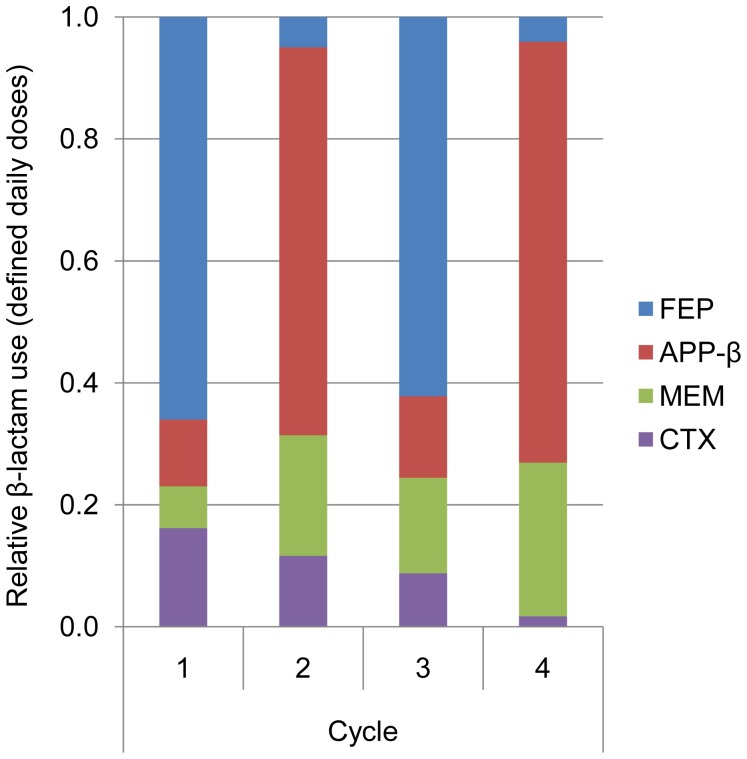
β-lactam use in cycle periods. Relative distribution of antibiotic use (defined daily doses) among the major β-lactams: cefepime (FEP), antipseudomonal penicillin combinations (APP-β), meropenem (MEM), and ceftriaxone or cefotaxime (CTX) use in the combined Units (n = 1987).

There were four consecutive cycles in Unit 1 (Westmead, WM), beginning in April 2004 (cefepime), August (APP-β), December (cefepime), and April 2005 (APP-β), respectively (Figure 1), and three in Unit 2 (Royal Brisbane and Women’s Hospital, RBWH), beginning in August 2004 (APP-β), December 2004 (cefepime) and April 2005 (APP-β). For a short period (February to March 2005), nationwide shortages of piperacillin/tazobactam (TZP) meant substitution with ticarcillin/clavulanate (TIM) as the APP-β, as these agents are generally regarded as closely comparable in their antibacterial spectra [13]. Hereafter the term APP-β is used for both.

Of the 1987 admissions to both ICUs in this period, 1194 were for at least 48 hours. Of these, 286 were admitted within 48 h of hospital arrival and received only cefepime or APP-β, or no antibiotics at all, by the end of their first week in ICU. In 206 of these (72%), late clinical followup and microbiological surveillance (see below) at all timepoints was complete. This group represents patients most clearly and directly subject to cycle-specific antibiotic influences. Data collected included age, sex, admission Acute Physiology and Chronic Health Evaluation (APACHE II) score, reason for admission, surgical and other interventions, ventilation and dialysis days, presence of central venous catheters, dialysis catheters, hospital and ICU length of stay (LOS), mortality, detailed antibiotic treatment, and microbiological information (Table 1). The standardised APACHE score remains the premier predictor of ICU and hospital mortality for ICU admissions with high predictive value [14] and high inter-rater reliability [15]. APACHE II [16] incorporates a range of acute (temperature, mean arterial pressure, blood pH, heart and respiratory rate, arterial-alveolar oxygen gradient, serum Na, K, Creatinine, white blood count, haematocrit, Glasgow Coma Score, presence of acute renal failure) and chronic indicators (age, hepatic failure, encephalopathy, cirrhosis, portal hypertension, heart failure, dialysis, chronic respiratory failure and respiratory conditions, and immunosuppression including leukaemia, lymphoma or AIDS). Antibiotic use, recorded as numbers of patients treated and as defined daily doses (DDD), included days on which the antibiotic regimen started and ended. Antibiotic doses were adjusted up and down from the standard guidelines by the duty intensive care specialist on the basis of usual pharmacokinetic considerations (renal and hepatic function, dialysis, volumes of distribution, protein binding) in individual patients, but antibiotic levels in blood and tissues were routinely measured only for aminoglycosides and vancomycin. All antibiotics, including vancomycin and gentamicin, were otherwise dosed and monitored according to standard Australian guidelines [13]. Cefepime dosing was recommended at 6 g/day (2 g every 8 h) for severe infection, and piperacillin 4 g plus tazobactam 500 mg (TZP 4.5 g) every 8 h or ticarcillin 3 g/clavulanate 100 mg (TIM 3.1 g) every 6 h for an average person with normal renal function.

**Table 1:** Patient characteristics.

Characteristic		Unit 1 (n = 1135)	Unit 2 (n = 852)	p^c^	All admissions (n = 1987)
Age (years) median (IQR)		59.5 (41.1–71.6)	57.8 (40.1–70.9)	0.203	58.7 (40.8–71.3)
Male, n (%)		690 (60.8)	522 (61.3)	0.433	1212 (61.0)
ICU LOS (days) median (IQR)		3.2 (1.5–7.3)	2.2 (1.0–6.4)	<0.001	2.8 (1.2–6.9)
HOS LOS (days) median (IQR)		17.5 (6.6–39.0)	16.3 (7.1–32.7)	0.529	17.0 (7.0–36.1)
APACHE II median (IQR)		20.0 (14.0–26.0)	18.0 (13.0–24.8)	0.005	19.0 (13.0–25.0)
Admission from community <48 h, n (%)		664 (58.5)	613 (71.9)	<0.001	1277 (64.3)
Admission category:	Trauma/surgical, n (%)	650 (57.3)	472 (55.4)	0.411	1122 (56.5)
	Medical, n (%)	485^a^ (42.7)	380 (44.6)	0.411	865 (43.5)
ICU Readmission, n (%)		47 (4.1)	45 (5.3)	0.138	92 (4.6)
Multiple ICU Readmissions, n (%)		19 (1.7)	10 (1.2)	0.234	29 (1.5)
Operative Intervention, n (%)		314 (27.7)	540^b^ (63.4)	<0.001	854 (43.0)
Intercranial drain or monitor, n (%)		56 (4.9)	74 (8.7)	0.001	130 (6.5)
Intercostal Drain, n (%)		40 (3.5)	80 (9.4)	<0.001	120 (6.0)
Nasogastric catheter, n (%)		864 (76.1)	657 (77.1)	0.323	1521 (76.5)
Endotracheal Tube or Tracheostomy, n (%)		842 (74.2)	629 (73.8)	0.448	1471 (74.0)
Urinary catheter, n (%)		913 (80.4)	819 (96.1)	<0.001	1732 (87.2)
Arterial catheter, n (%)		889 (78.3)	797 (93.5)	<0.001	1686 (84.9)
Central venous catheter, n (%)		836 (73.7)	594 (69.7)	0.3	1430 (72.0)
Other vascular (dialytic) catheter, n (%)		95 (8.4)	86 (10.1)	0.207	181 (9.1)
Dialysis, n (%)		92 (8.1)	84 (9.9)	0.176	176 (8.9)
ICU Mortality, n (%)		137 (12.1)	112 (13.1)	0.258	249 (12.5)
Hospital Mortality, n (%)		165 (14.5)	136 (16.0)	0.208	301 (15.1)

Patient characteristics of all admissions.

a includes haematology/marrow transplant (∼1% of admissions to both units);

b includes tracheostomies;

c Mann-Whitney U test or Chi-Squared analysis.

### Microbiological Analysis

Positive cultures from all clinically indicated specimens submitted to the diagnostic laboratory were identified. Respiratory tract isolates were reported as significant if a pathogen was identified as sufficiently numerous in semi-quantitative assays (≥10^4^ cfu/mL in non-bronchoscopic or directed bronchoalveloar lavage) or was predominant in a purulent sputum specimen without significant epithelial contamination evident (<10 epithelial cells per high-power field) on microscopy. Isolates were also deemed significant if grown from normally sterile sites (e.g., bloodstream) or if predominant in the specimen on microscopy and culture (e.g., deep tissues) or if pyuria was present and >10^6^ cfu/mL of a single organism was grown from urine. The reporting laboratory must also have issued full identification and susceptibility data on the basis of established standard laboratory criteria in both centres, in accordance with established definitions [17] by prior agreement between investigators in both units.

All specimens were given unique numbers and de-identified before processing. Surveillance samples (perineal swab and endotracheal aspirate) obtained on admission and twice weekly thereafter were submitted to the centralised reference laboratories serving each unit (Unit 1: Centre for Infectious Diseases and Microbiology (CIDM) Laboratory Services at Westmead Hospital, Sydney; Unit 2: Queensland Pathology at the Royal Brisbane and Women’s Hospital, Brisbane), and inoculated into nutrient broth overnight, resuspended in 20% glycerol in nutrient broth (GNB, Difco) and stored at -80°C for transfer to CIDM. A loopful of each frozen sample was resuspended in nutrient broth at 37°C for 90 min aerobically before inoculation onto solid media. *Pseudomonas aeruginosa* was confirmed by growth on Pseudomonas Isolation Agar (Becton Dickinson) [18] and oxidase activity (Oxoid), and methicillin-resistant *Staphylococcus aureus* (MRSA) by growth on Brilliance MRSA Agar (Oxoid) [19] with confirmatory testing using the BactiStaph reagent (Remel) [20]. MRSA isolates were subtyped by *Sma*I PFGE as previously described [21] and analysed for relatedness as per standard criteria [22]. *Enterobacteriaceae* were detected by their morphology on chromogenic agar (CHROMagar Orientation) [23], and tested for growth in the presence of ticarcillin/clavulanate (128/2 µg/mL), gentamicin (10 µg/mL) or cefepime (64 µg/mL) incorporated into the agar. Colonies with the typical appearance of *E. coli* or of *Klebsiella/Enterobacter/Serratia/Citrobacter spp*. on CHROMAgar, in accordance with the Australian National Accreditation Testing Authority approved methodology in each laboratory and with the media manufacturer’s guidelines, were designated “*Enterobacteriaceae*” after testing to exclude oxidase activity. All clinical isolates of *P. aeruginosa* and all TIM-resistant *P. aeruginosa* isolates from surveillance samples, as well a random subset of TIM-resistant *Enterobacteriaceae*, were also tested against TZP. No significant differences were found in antimicrobial susceptibility so TIM was retained as the APP-β resistance marker. Unless otherwise specified in the text, ‘resistant’ or ‘resistance’ refers to APP-β, gentamicin and/or cefepime, in the above concentrations. All MRSA isolates were resistant to these three antibiotics, but *P. aeruginosa* isolates were specified as ‘resistant’ if resistant to any.

### Statistical Analysis

Acquisition of infection and/or colonisation by antibiotic-resistant bacteria and by *P. aeruginosa* and methicillin-resistant *S. aureus* was the primary outcome for analysis. Each significant isolate and each antibiotic-resistant colonisation was counted only once per admission. Data were analysed separately for both hospitals before being pooled, as the differences between the two Units in terms of casemix and basic demographics were minor (see Table 1). Statistical analysis was performed using SPSS (v18, SPSS Inc.). Proportions were compared using the Mann-Whitney U, one-way analysis of variance (ANOVA) or Chi-Squared tests, as specified. Analysis of multiple predictors for ICU clinical and bacteriological outcomes was performed using step-wise backwards logistic regression models, considering only those factors significant at p≤0.10 in univariate analysis, and retaining only factors significant at p≤0.05 in the final model.

**Figure 2:**
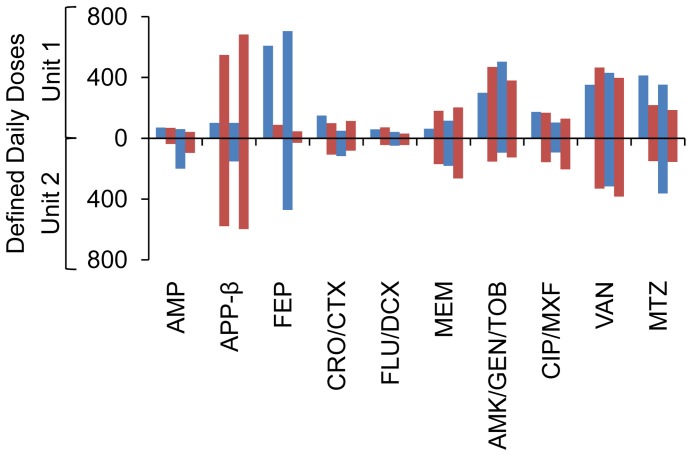
Antibiotic usage. Total defined daily doses in each cycle (n = 1987) in Unit 1 (above line) and Unit 2 (below line); black bars, FEP cycles; white bars, APP-β cycles; AMP, ampicillin; APP-β, antipseudomonal penicillin combinations; FEP, cefepime; CRO, ceftriaxone; CTX, cefotaxime; FLU, flucloxacillin; DCX, dicloxacillin; MEM, meropenem; AMK, amikacin; GEN, gentamicin; TOB, tobramycin; CIP, ciprofloxacin; MXF, moxifloxacin; VAN, vancomycin; MTZ, metronidazole.

## Results

### The Study Population

Admissions to Unit 1 (n = 1135) and Unit 2 (n = 852) were similar in casemix and other key characteristics (Table 1), and were combined for most analyses. Illness severity was moderately high, with median APACHE II scores of 20 (IQR 14–26) vs. 18 (IQR 13–25), ICU mortality rates of 12.1% vs. 13.1% and in-hospital mortality of 14.5% vs. 16.0% for Units 1 and 2, respectively. Predictors of mortality within the set of all admissions (n = 1987) by multivariate analysis were age>65 (OR 1.45; 95% CI 1.10–1.90), endotracheal intubation for mechanical ventilation (OR 1.59; 95% CI 1.14–2.23) and renal failure requiring dialysis (OR 2.92; 95% CI 1.89–4.53), all p<0.01. Surgical admissions were associated with a relatively lower mortality risk (OR 0.49; 95% CI 0.37–0.65; p<0.001).

### Antibiotic Homogeneity and Main Outcomes

Antibiotic homogeneity was high: cycle-specified drug comprised >60% of major β-lactam antibiotic use in respective on-cycles and <15% in off-cycles (Figure 1). Other antibiotic use was generally consistent between cycles (Figure 2). Unit 2 had significantly more admissions from the community (71.9 vs 58.5%) and a higher incidence of urinary catheterisation (96.1 vs 80.4%), but there were no other important differences in major demographic or casemix variables, and no significant differences in infection rates, mortality or length of stay, either between cycles or from beginning to end-study, when data from both Units were considered together (Table 2 and 3). The final cycle had the most patients, the highest proportion mechanically ventilated (Table 2), the highest antibiotic use, and the highest treatment intensity (cycle 1∶2449 DDD/1965 patient days, ratio 1.25; cycle 2∶4656/3737, 1.25; cycle 3∶5012/3656, 1.37; cycle 4 4830/3362, 1.44; Table 4). Despite this, the proportion of admissions complicated by infection in the last (APP-β) cycle was unchanged in Unit 1, and reduced in Unit 2 when compared to previous cycles (Table 3).

**Table 2:** Patient characteristics in each cycle.

Characteristic		Cycle 1 (n = 284)	Cycle 2 (n = 571)	Cycle 3 (n = 557)	Cycle 4 (n = 575)
Age (yrs); Median (IQR)		60.5 (14.0–72.0)	60.5 (39.9–71.9)	55.5 (39.6–71.7)	58.5 (40.9–70.0)^ d^ *
Male, n (%)		160 (56.3) ^a^ *	368 (64.4)	336 (60.3)	348 (60.5)
ICU LOS (days); Median (IQR)		3.0 (751.0–7.3)	2.6 (1.0–6.8)	2.9 (1.8–7.5)	2.8 (1.4–6.8)
Hospital LOS (days); Median (IQR)		17.0 (7.0–37.9)	16.5 (6.9–35.7)	16.8 (7.3–34.8)	17.7 (6.0–37.2)
Age >65 years, n (%)		112 (39.4)	229 (40.1)	193 (34.6)	195 (33.9)
APACHE II; Median (IQR)		20.0 (1.5–26.0)	18.0 (13.0–26.0)	19.0 (13.0–25.5)	18.0 (13.0–24.0)
Admission from community <48 h, n (%)		145 (51.1) ^a^ ***	372 (65.1)	377 (67.7)	384 (66.8)^ d^ ***
Admission category:	Trauma/surgical, n (%)	159 (56.0)	328 (57.4)	322 (57.8)	313 (54.4)
	Medical, n (%)	125 (44.0)	243 (42.6)	235 (42.2)	262 (45.6)
ICU Readmission, n (%)		14 (4.9)	30 (5.3)	23 (4.1)	25 (4.3)
Multiple ICU Readmissions, n (%)		6 (2.1)	15 (2.6)^ b^ **	3 (0.5)	5 (0.9)
Operative Intervention, n (%)		76 (26.8)^ a^ ***	257 (45.0)	246 (44.2)	275 (47.8)^ d^ ***
Intercranial drain or monitor, n (%)		19 (6.7)	36 (6.3)	31 (5.6)	44 (7.7)
Intercostal Drain, n (%)		15 (5.3)	37 (6.5)	38 (6.8)	30 (5.2)
Nasogastric catheter, n (%)		196 (69.0)	4.9 (71.6)	426 (76.5)^ c^ ***	490 (85.2)^ d^ ***
Endotracheal Tube or Tracheostomy, n (%)		205 (72.2)	391 (68.5)	401 (72.0)^ c^ ***	474 (82.4)^ d^ ***
Urinary catheter, n (%)		221 (77.8)^ a^ *	480 (84.1)	486 (87.3)^ c^ ***	545 (94.8)^ d^ ***
Arterial catheter, n (%)		215 (75.7)^ a^ **	479 (83.9)	471 (84.6)^ c^ *	521 (90.6)^ d^ ***
Central venous catheter, n (%)		217 (76.4)^ a^ **	383 (67.1)	394 (70.7)	436 (75.8)
Other vascular (dialytic) catheter, n (%)		14 (4.9)	40 (7.0)	44 (7.9)	44 (7.7)
Dialysis, n (%)		13 (4.6)	25 (4.4)	38 (6.8)	32 (5.6)
ICU Mortality, n (%)		47 (16.5)	66 (11.6)	56 (10.1)	80 (13.9)
Hospital Mortality, n (%)		63 (22.2)^ a^ ***	72 (12.6)	67 (12.0)^ c^ *	99 (17.2)

a Significant difference between cycle 1 (Unit 1 only) and 2;

b between cycle 2 and 3;

c between cycle 3 and 4;

d between cycle 1 and 4;

* p<0.05;

** p<0.01;

*** p<0.001.

**Table 3:** MRSA and *P. aeruginosa* infection and colonisation after ICU admission.

			all SI	*P. aerug.* (SI)		MRSA (SI)		*P. aerug.* (CI)		MRSA (CI)	
Unit	Cycle	Adm.	(rate)	n	(rate; % SI)	n	(rate; % SI)	n/tested	(rate)	n/tested	(rate)
1	1 FEP	180	34.44	14	7.78	24	13.33	18/51		26/51	
(n = 743)	2 APP-β	181	36.46	7	3.87	15	8.29	21/49		23/49	
	3 FEP	182	35.71	13	7.14	28	15.38*	23/57		23/57	
	4 APP-β	200	33.00	7	3.50	18	9.00*	22/52		20/52	
	**All FEP**	362	35.08	27	**(7.46; 21.3)**	52	**(14.36; 40.1)**	41/108	(38.9)	49/108	(48.1)
	**All APP-β**	381	34.65	14	**(3.67** * **; 10.6)**	33	**(8.66** * **; 25.0)**	43/101	(42.6)	43/101	(42.6)
2	2 APP-β	144	80.15	20	13.89	8	5.56	6/31		0/31	
(n = 451)	3 FEP	154	77.12	24	15.58	8	5.19	3/26		0/26	
	4 APP-β	153	55.56*	13	8.50	8	5.23	5/20		1/20	
	**All FEP**	154	77.12	24	**(15.58; 20.3)**	8	**(5.19; 6.8)**	3/26	(11.5)	0/26	(0.0)
	**All APP-β**	297	67.50*	33	**(11.11; 17.5)**	16	**(5.39; 8.5)**	11/51	(21.6)	1/51	(2.0)

Significant isolates (SI) in patients admitted for more than 48 h (n = 1194;) compared with commensal isolates (CI) from those admitted to ICU within 48 h of hospital arrival and received only cefepime or APP-β, or no antibiotics at all, by the end of their first week in ICU (n = 286). FEP, cefepime; APP-β, antipseudomonal penicillin/β-lactamase inhibitor combinations;

* indicates significance (p-value <0.05) relative to the previous cycle, by Chi-Squared analysis. Rate: patients positive per 100 admissions; % SI: rate per 100 significant isolates.

**Table 4:** Antibiotic prescribing trends in cycles.

	Cycle 1 n (days)				Cycle 2 n (days)				Cycle 3 n (days)				Cycle 4 n (days)			
Antibiotic	First	Mid		Last	First	Mid		Last	First	Mid		Last	First	Mid		Last
PEN	1 (2)	0 (0)		0 (0)	7 (22)	8 (56)		5 (26)	1 (1)	8 (28)		5 (11)	4 (19)	11 (46)		13 (59)
AMP	9 (35)	6 (14)		9 (22)	12 (48)	12 (19)		13 (39)	25 (88)	23 (112)		23 (60)	19 (76)	10 (46)		5 (15)
CEF/CFZ	20 (49)	24 (69)		17 (31)	44 (136)	30 (79)		46 (121)	37 (99)	43 (136)		41 (115)	30 (83)	34 (127)		35 (123)
FLU/DCX	1 (6)	5 (21)		6 (32)	10 (36)	9 (34)		8 (46)	5 (12)	9 (53)		6 (25)	8 (31)	4 (21)		7 (23)
CAZ	0 (0)	0 (0)		0 (0)	1 (1)	0 (0)		1 (3)	1 (3)	0 (0)		0 (0)	3 (20)	2 (3)		1 (2)
CTX/CRO	15 (54)	17 (48)		14 (47)	27 (105)	15 (59)		15 (42)	23 (91)	10 (24)		15 (51)	21 (90)	18 (52)		20 (52)
FEP	21 (**133**)	38 (**273**)		26 (**202**)	5 (31)	3 (31)		3 (26)	68 (**409**)	68 (**425**)		57 (**342**)	7 (50)	3 (13)		3 (12)
APP-β	4 (29)	6 (49)		6 (23)	72 (**423**)	63 (**357**)		70 (**346**)	23 (112)	18 (66)		15 (75)	70 (**440**)	66 (**300**)		82 (**539**)
MEM	7 (29)	3 (11)		4 (23)	21 (167)	14 (56)		17 (127)	11 (65)	17 (139)		13 (92)	26 (261)	13 (91)		16 (115)
AMK	1 (5)	1 (4)		3 (3)	1 (2)	0 (0)		1 (2)	1 (10)	0 (0)		2 (2)	0 (0)	2 (8)		1 (3)
GEN	21 (105)	26 (112)		23 (70)	46 (190)	50 (211)		38 (167)	40 (139)	38 (169)		55 (269)	38 (149)	39 (119)		47 (197)
TOB	0 (0)	0 (0)		0 (0)	0 (0)	6 (46)		2 (4)	1 (2)	1 (3)		2 (4)	4 (27)	2 (2)		1 (1)
MTZ	23 (146)	32 (165)		28 (102)	24 (142)	27 (135)		21 (91)	52 (220)	52 (281)		49 (215)	31 (117)	34 (127)		22 (96)
CIP	3 (15)	5 (28)		1 (2)	13 (114)	6 (41)		8 (62)	8 (32)	3 (10)		8 (64)	13 (115)	10 (44)		10 (62)
MXF	8 (36)	7 (57)		8 (36)	15 (82)	2 (12)		4 (14)	6 (27)	9 (43)		6 (22)	7 (38)	6 (43)		7 (32)
TEC	0 (0)	0 (0)		1 (3)	1 (7)	1 (5)		2 (10)	0 (0)	0 (0)		0 (0)	0 (0)	0 (0)		0 (0)
VAN	26 (122)	29 (134)		21 (96)	53 (323)	40 (224)		41 (249)	45 (243)	43 (225)		48 (278)	46 (322)	49 (173)		56 (285)
LZD	0 (0)	0 (0)		0 (0)	0 (0)	1 (34)		0 (0)	2 (10)	0 (0)		3 (64)	1 (42)	3 (7)		1 (8)
SXT	0 (0)	1 (6)		0 (0)	0 (0)	1 (2)		1 (5)	1 (1)	3 (27)		2 (18)	5 (31)	4 (27)		4 (46)
CST	0 (0)	0 (0)		0 (0)	0 (0)	1 (46)		0 (0)	0 (0)	0 (0)		0 (0)	0 (0)	0 (0)		0 (0)
Total antibiotic days/patient days (ratio)			2449/1965 (1·25)				4656/3737 (1·25)				5012/3656 (1·37)				4830/3362 (1·44)	

Number of patients receiving specified antibiotic in each cycle, n, and total patient days (days).

First: first third of cycle; mid: middle third of cycle; last: last third of cycle.

PEN, penicillin; AMP, ampicillin; CEF, cephalothin; CFZ, cefazolin; FLU, flucloxacillin; DCX, dicloxacillin; CAZ, ceftazidime; CTX, cefotaxime; CRO, ceftriaxone; FEP, cefepime; APP-β, antipseudomonal penicillin combinations; MEM, meropenem; AMK, amikacin; GEN, gentamicin; TOB, tobramycin; MTZ, metronidazole; CIP, ciprofloxacin; MXF, moxifloxacin; TEC, teicoplanin; VAN, vancomycin; LZD, linezolid; SXT, co-trimoxazole; CST, colistin (polymixin E).

### Risk of Infection by Resistant Bacteria is Higher in Cefepime Cycles

In order to determine the impact of the two different β-lactam regimens, we compared the risk of infection and/or colonisation with either MRSA or *P. aeruginosa* or other antibiotic resistant bacteria. Two hundred and twenty-three admissions in all were complicated at some stage by resistant infection. Of all infecting bacteria resistant to any of the three first-line antibiotics, APP-β (isolated from n = 121, 54.3% of admissions) and gentamicin resistance (132, 59.2%) were most common. Cefepime resistance was rare among *Enterobacteriaceae* (2, 0.9%) and *P. aeruginosa* (11, 4.9%). A cefepime-resistant infection complicated only 32 (14.3%) admissions. Forty-one (18.4%) of the 223 admissions complicated by resistant infection were complicated by antibiotic-resistant *P. aeruginosa* and 81/223 (36.3%) by MRSA.

Although there was no difference in the proportion of admissions complicated by any infection at all, the proportion of admissions complicated by antibiotic-resistant infection was more than twice as high in cefepime cycles as in APP-β cycles (164.1 vs 74.2 per 1000 admissions; p<0.001). MRSA infection complicated significantly more cefepime than APP-β cycle admissions (116.3 vs. 72.2/1000 admissions; p 0.01), and this was also evident between individual cycles (Table 3). *P. aeruginosa* infection (any/all, including gentamicin and APP-β susceptible) complicated a greater proportion of admissions in cefepime than APP-β cycles (Table 3), and this was statistically significant in Unit 1, although analysis according to resistance phenotype was not statistically meaningful. A similar trend in both Units for increased infection by (any/all) *P. aeruginosa* in cefepime cycles (cefepime vs APP-β: 98.8 vs 69.3/1000; Table 3) was not statistically significant (p 0.07).

Fourteen cefepime-cycle admissions were complicated by antibiotic-resistant bacteraemia compared to nine APP-β-cycle admissions although the difference between these small numbers was not significant (16.6 vs 7.9 per 1000 admissions; p 0.089). About a third of all bacteraemias (10/35, Unit 1; 5/12, Unit 2) were due to *Enterobacteriaceae* but few were antibiotic-resistant and these were not further analysed.

### Risk of Colonisation by Resistant Bacteria is higher in Cefepime Cycles

**Figure 3:**
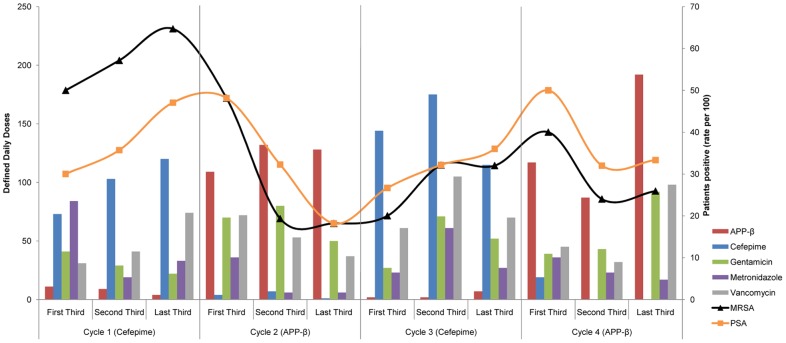
Antibiotic treatment and *P. aeruginosa* and MRSA colonisation rates. Total defined daily doses of selected antibiotics in cycle thirds in patients admitted to ICU within 48 h of hospital arrival and received only cefepime or APP-β, or no antibiotics at all, by the end of their first week in ICU (vertical bars; left axis), with incidence rates (solid lines; right axis) of unique isolation of MRSA (triangles) and *P. aeruginosa* (squares) from commensal sites (ie. colonisation rates, per 100 admissions).

Increased infection in those exposed to cefepime should be associated with increased colonisation rates. We identified admissions direct from the community who received either specified β-lactam (and no off-cycle β-lactam) for >48 hours before sampling in the first week of admission, or no antibiotics at all (60/152, 39% of APP-β cycle admissions; 58/134, 43% of cefepime cycle admissions). This subset (n = 286), with higher admission APACHE II scores and ICU length of stay (Table S1), most accurately reflects antibiotic influence. When data were pooled and compared by cycle type, there were no significant differences in overall proportions of admissions in which MRSA or *P. aeruginosa* was isolated from surveillance samples (Table 3). However, when the first, middle and final thirds of each cycle are plotted to account for ‘washout’ from the preceding cycle, a trend is suggested (Figure 3), and many of the relative proportions are significantly different when directly compared (Table 5).

**Table 5:** Colonisation of patients with MRSA or *P. aeruginosa* within portions of cycles.

	MRSA						*P. aeruginosa*					
	FEP			APP-β			FEP			APP-β		
Portion of cycle	n/tested	rate	p	n/tested	rate	p	n/tested	rate	p	n/tested	rate	p
First Half	21/68	0.31	0.076	24/70	0.34	0.211	15/68	0.22	0.006	32/70	0.46	0.018
Second Half	31/66	0.47		20/82	0.24		30/66	0.45		22/82	0.27	
First third	16/50	0.32		21/47	0.45		14/50	0.28		23/47	0.49	
Second Third	17/42	0.4	0.192	12/56	0.21	0.018	14/42	0.33	0.21	18/56	0.32	0.023
Last Third	19/42	0.45		11/49	0.22		17/42	0.4		13/49	0.27	

p-values indicate significance between portions of cycles for each organism, by Chi-Squared analysis. FEP, cefepime; APP-β, antipseudomonal penicillin/β-lactamase inhibitor combinations. Rate: proportion of patients positive.

Sampling was complete at all time points in 206 of these 286 admissions. Of these 206, 63 (30.6%) developed perineal and/or endotracheal colonisation by antibiotic-resistant bacteria in the first week. Of 63 newly colonised admissions identified, 18 (28.6%) acquired MRSA, 36 (57.1%) acquired *P. aeruginosa* and 44 (69.8%) acquired resistant *Enterobacteriaceae*. Backwards stepwise logistic regression analysis revealed cefepime treatment in a cefepime cycle as the sole independent predictor of acquiring resistant bacteria at surveillance sites (OR 2.51; 95% CI 1.35–4.66; p 0.003). Those treated with cefepime were significantly more likely to acquire MRSA (OR 3.612; 95% CI 1.33–9.79; p 0.012) or antibiotic-resistant *Enterobacteriaceae* (OR 3.184; 95% CI 1.589–6.380; p 0.001). New acquisition of (any) *P. aeruginosa* was also twice as common in the cefepime than APP-β -treated admissions, but this was not quite statistically significant at 95% confidence levels (OR 2.02; 95% CI 0.96–4.23; p 0.063). The only factor associated with reduced risk of antibiotic-resistant colonization was an operative intervention (OR 0.049; 95% CI 0.241–0.990; p 0.047). Although APP-β treatment did not independently predict reduced risk, cefepime treatment is independently predictive of increased risk of resistance acquisition in multivariate analysis.

### Diversity of MRSA Subtypes

Clonal MRSA outbreaks that were coincidental with the antibiotic cycle changes were excluded as an explanation for these findings. Standardised methodology [22] revealed 35 distinct MRSA pulsotypes, within 17 clearly unrelated groups (<84% similarity; Dice coefficient, represented by UPGMA, 0.5% optimization and 1.0% tolerance) that were distributed throughout the study. The majority of these (∼75%) were members of the dominant clonal complex in Australia (Aus 2/3; multi-locus sequence type 239), with complexes 93, 30, 22, 5 and 36 also identified. Diversity was similar in each cycle: cycle 1 (cefepime; Unit 1 only) had 7 PFGE variants of ≤95% similarity, cycle 2 (APP-β) had 10 such variants, cycle 3 (cefepime) had 13 variants and cycle 4 (APP-β) had 11 variants. The largest group of closely related isolates (≥95% similarity) spanned two cycles over a 6-month period (Mar - Sept 2004). This group comprised only 20% of isolates in that period, were interspersed with less closely related isolates, and still occurred sporadically a year later (Figure S1). These data indicate that different MRSA clones appeared throughout the study, as expected for a general selection effect.

## Discussion

The model of antibiotic cycling is part of normal ICU practice, and in similar studies [24], [25] a waiver of consent was granted for the same reasons as apply here. Here, we show that cefepime exposure is the strongest single independent risk factor for colonization and infection of intensive care patients by antibiotic-resistant bacteria. We found no evidence of a cumulative or persistent effect, in keeping with a similar TZP/cefepime cycling study in transplant patients that reported preservation of antibiotic susceptibility among Gram-negative bacteria [26].

We found no support for mathematical models that identify antibiotic homogeneity as a specific resistance driver [27]. This conflict may be explained by the necessity to employ simplified assumptions about biological mechanisms [28]. Previous studies of antibiotic cycling often compared antibiotics with vastly different mechanisms of action and ecological effects, and often used extremely short cycles [29] in spite of evidence that antibiotic effects last for many weeks and, for some elements of the microflora, months to years [30]. Activity of cefepime against relevant pathogens was superior to that of APP-β *in vitro* but APP-β seem to have less adverse effects on the microflora than third-generation cephalosporins [27], [30]. Antibiotic levels in tissues or gut were not defined in this study, but biliary penetration of cefepime is relatively poor compared to that of the antipseudomonal penicillins [31]. Unlike cefepime, the activity of antipseudomonal penicillins such as TZP against some of the *Enterobacteriaceae* with class 1 cephalosporinases is dependent on the tazobactam component, which is relatively less excreted in bile than piperacillin [32], while both the gut penetration and the *in vitro* activity of piperacillin is superior to that of cefepime against the major anaerobic pathogens [31]. Metronidazole (MTZ) was commonly added to cefepime by prescribers in this study and differences in activity (between the cefepime/MTZ combination and the antipseudomonal penicillin/β-lactamase inhibitor combinations) against commensal anaerobes may also be relevant. In the mouse model, disruption of the gut microflora by a third-generation cephalosporins does not spontaneously revert, unlike the effects of a penicillin (ampicillin) [33]. An association between third-generation cephalosporins [34] and cefepime [35] and increased prevalence of resistant pathogens such as *Clostridum difficile* [36], [37], *P. aeruginosa* [38], antibiotic-resistant *Enterobacteriaceae* [39] and MRSA [39], [40], [41] is well described. Lasting effects on the microflora, as reported for other antibiotics [8], [9], cannot be excluded in the absence of long-term follow-up after ICU discharge but we found no evidence of cumulative risk. Antibiotic resistance rates overall fell to baseline in APP-β cycles, presumably as cefepime-exposed admissions were discharged from ICU. MRSA and *P. aeruginosa* infection rates also rose and fell according to cycle, and underlying colonisation rates appeared to behave in the same way.

**Table 6:** Infection rates.

Source	Organism	Unit 1	Rate	Unit 2	Rate	p^e^
**blood**	MRSA	6	5.3	1	1.2	
	MSSA	6	5.3	1	1.2	
	*Enterococcus*	8	7.1	3	3.5	
	*Streptococcus*	3	2.6	–	–	
	CNS^a^	89	78.4	37	43.4	
	*E. coli*	3	2.6	1	1.2	
	*Klebsiella*	2	1.8	1	1.2	
	*ESCPM* b	5	4.4	3	3.5	
	*P. aeruginosa*	–	–	2	2.4	
	bacterial total	122	109.3	49	57.5	
	(excl. CNS)	35	**30.8**	12	**14.1**	0.022
**fluids and**	MRSA	2	1.8	–	–	
**tissues** c	MSSA	2	1.8	–	–	
	*Enterococcus*	–	–	1	1.2	
	CNS	14	12.3	17	20.0	
	*E. coli*	4	3.5	3	3.5	
	*Klebsiella*	1	0.9	2	2.4	
	*ESCPM*	–	–	4	4.7	
	*P. aeruginosa*	5	4.4	3	3.5	
	*Stenotrophomonas.*	1	0.9	–	–	
	bacterial total	29	25.6	30	36.2	
	(excl. CNS)	15	**13.2**	13	**15.3**	0.705
**urine**	MRSA	–	–	–	–	
	MSSA	–	–	1	1.2	
	*Enterococcus*	9	7.9	2	2.4	
	CNS	–	–	1	1.2	
	*E. coli*	8	7.0	8	9.4	
	*Klebsiella*	4	3.5	2	2.4	
	*ESCPM*	7	6.2	3	3.5	
	*P. aeruginosa*	8	7.0	4	4.7	
	*NF* d	1	0.9	1	1.2	
	bacterial total	37	32.6	22	25.8	
	(excl. CNS)	37	**32.6**	21	**24.6**	0.347
**respiratory**	MRSA	40	35.2	8	9.4	
	MSSA	24	21.1	53	62.2	
	*Enterococcus*	–	–	1	1.2	
	*S. pneumoniae*	–	–	5	5.9	
	*E. coli*	11	9.7	21	24.6	
	*Klebsiella*	19	16.7	49	57.5	
	*ESCPM*	28	24.7	39	45.8	
	*P. aeruginosa*	32	28.2	28	32.9	
	*NF*	11	9.7	47	55.2	
	*Haemophilus*	–	–	17	20.0	
	bacterial total	165	145.4	268	314.6	
	(excl. NF)	154	**135.7**	221	**259.4**	<0.001

Unique infections in all patients admitted to ICU (n = 1987) are shown in absolute numbers and in rates and a two-tailed test applied to compare the rates per 1000 admissions;

a includes all coagulase-negative staphylococci, and other organisms deemed to be contaminants eg. *Corynebacterium* spp. and *Micrococcus* spp.;

b ESCPM: *Enterobacter, Serratia, Citrobacter, Proteus, Morganella* spp.;

c includes surgical specimens and drainage procedures;

d NF: glucose non-fermenting Gram-negative bacilli, including *Acinetobacter* spp., *Pseudomonas* spp. *Stenotrophomonas* spp., etc.

e Chi-Squared analysis.

Cefepime was associated with increased *P. aeruginosa* colonisation and infection, and significantly so for combined FEP cycles (27/362) vs APP-β cycles (14/381) in Unit 1 (p 0.024). Ratios between bacteraemia and respiratory infection and deep isolates were similar between Units for MRSA and for methicillin-sensitive *S. aureus* (MSSA), although respiratory infection was more commonly reported in Unit 2 in general, and this included Gram-negative pathogens as well as *S. aureus* (Table 6). Indeed, the overwhelming source of all significant isolates in Unit 2 was respiratory, at which site the attribution of significance to pathogens such as MRSA and *P. aeruginosa* is problematic. Nevertheless, rates of infection in fluids and tissues were similar between Units, as expected given the similar casemix and acuity. More frequent reporting of *P. aeruginosa* in Unit 2 (a burns referral centre) may have arisen indirectly, through greater awareness of the pathogen, and this would be another potential confounder. Any bias introduced by relatively higher incidence of community admissions to Unit 2 introduces would be expected to operate to reduce rates of nosocomial *Pseudomonas* infection but Unit 2 also reported a higher rate of urinary catheterization which, combined with the possible variation in respiratory tract reporting, may relatively increase apparent *Pseudomonas* infection rates. In any case however, the fact that infection was less common in Unit 2 than Unit 1 in almost every site including the bloodstream and that only Unit 2 experienced *P. aeruginosa* bacteraemias suggests that the difference is real. This is difficult to further clarify, although it is reasonable to assume that any attribution of undue significance to respiratory *P. aeruginosa* in Unit 2 and/or under-attribution in Unit 1 would be unaffected by cycling and remain relatively constant from cycle to cycle.

Neither length of stay nor mortality changed significantly overall in this study, despite the known consequences of antibiotic-resistant infection [42]. In Unit 1, with the highest MRSA incidence, MRSA infection complicated 8.7% of APP-β cycle admissions, with more than a 60% increase (to 14.4%) in cefepime cycle admissions. If MRSA infection doubles mortality risk [43], a cohort of at least 1000 cefepime-treated patients would be required to detect a difference over a baseline mortality of 12%. A meta-analysis of 57 studies comparing cefepime with another β-lactam antibiotic in more than 3000 patients with sepsis reported an increased risk of all-cause mortality associated with cefepime not long after this study closed recruiting, the greatest difference being between TZP and FEP [12], [44]. These findings were later disputed [45] and then contradicted [46], but discrepancies between the original data and the unpublished data submitted by the drug sponsor [47] left the issue unresolved [48], [49]. Longer cycles or a larger study might have revealed statistically stronger differences. A disproportionate incidence of MRSA in Unit 1 and the absence of a second cefepime cycle in Unit 2 are weaknesses, but clonal MRSA outbreaks in Unit 1 do not provide an explanation. Overall antibiotic resistance was not detectably increased in our study by antibiotic cycling and independently obtained data from NSW Health also showed that MRSA acquisition rates did not increase in Unit 1 after the cycling study (Figure S2).

Our data show that cefepime therapy is associated with increased infection due to organisms resistant to the key antibiotics used for management of sepsis and septic shock in intensive care. Importantly, prescribing homogeneity *per se* does not appear to be a specific resistance driver. The ecological influences of cefepime seem to exceed that of all other risk factors in determining infection risk and may be relevant to unexplained mortality differences between cefepime and TZP in large studies of the critically ill. Failure to consider ecological effects in setting antibiotic policy may increase antibiotic resistance and even contribute to unintended mortality.

## Supporting Information

Figure S1 **Temporal distribution of MRSA pulsotypes within cycling period.** ST: Sequence type (letters indicate subgroups within STs at 95% identity). FEP: cefepime cycles; APP-β: antipseudomonal penicillin combination cycles.
(TIF)

Figure S2 **Monthly MRSA acquisition rates in Unit 1 before, during and after cycling (data from NSW Health Dept), shown as patients positive per 100 bed days.** FEP: cefepime cycles; APP-β: antipseudomonal penicillin combination cycle.
(TIF)

Table S1 **Patient characteristics of all admissions in which sampling was complete (n = 206).** ^a^Mann-Whitney U test or Chi-Squared analysis.
(DOC)

## References

[1] Kumar A, Haery C, Paladugu B, Kumar A, Symeoneides S (2006). The duration of hypotension before the initiation of antibiotic treatment is a critical determinant of survival in a murine model of *Escherichia coli* septic shock: association with serum lactate and inflammatory cytokine levels.. J Infect Dis.

[2] Kumar A, Roberts D, Wood KE, Light B, Parrillo JE (2006). Duration of hypotension before initiation of effective antimicrobial therapy is the critical determinant of survival in human septic shock.. Crit Care Med.

[3] de Smet AM, Kluytmans JA, Cooper BS, Mascini EM, Benus RF (2009). Decontamination of the digestive tract and oropharynx in ICU patients.. N Engl J Med.

[4] Oostdijk EA, de Smet AM, Blok HE, Thieme Groen ES, van Asselt GJ (2010). Ecological effects of selective decontamination on resistant Gram-negative bacterial colonization.. Am J Respir Crit Care Med.

[5] Kollef MH, Vlasnik J, Sharpless L, Pasque C, Murphy D (1997). Scheduled change of antibiotic classes: a strategy to decrease the incidence of ventilator-associated pneumonia.. Am J Respir Crit Care Med.

[6] Sandiumenge A, Diaz E, Rodriguez A, Vidaur L, Canadell L (2006). Impact of diversity of antibiotic use on the development of antimicrobial resistance.. J Antimicrob Chemother.

[7] Warren DK, Hill HA, Merz LR, Kollef MH, Hayden MK (2004). Cycling empirical antimicrobial agents to prevent emergence of antimicrobial-resistant Gram-negative bacteria among intensive care unit patients.. Crit Care Med.

[8] Brown EM, Nathwani D (2005). Antibiotic cycling or rotation: a systematic review of the evidence of efficacy.. J Antimicrob Chemother.

[9] Masterton RG (2005). Antibiotic cycling: more than it might seem?. J Antimicrob Chemother.

[10] Bergstrom CT, Lo M, Lipsitch M (2004). Ecological theory suggests that antimicrobial cycling will not reduce antimicrobial resistance in hospitals.. Proc Natl Acad Sci U S A.

[11] Levin BR, Bonten MJ (2004). Cycling antibiotics may not be good for your health.. Proc Natl Acad Sci U S A.

[12] Yahav D, Paul M, Fraser A, Sarid N, Leibovici L (2007). Efficacy and safety of cefepime: a systematic review and meta-analysis.. Lancet Infect Dis.

[13] Antibiotic Expert Group (2010). Therapeutic guidelines: antibiotic.. Melbourne: Therapeutic Guidelines Limited.

[14] Breslow MJ, Badawi O (2012). Severity scoring in the critically ill: part 1–interpretation and accuracy of outcome prediction scoring systems.. Chest.

[15] Kho ME, McDonald E, Stratford PW, Cook DJ (2007). Interrater reliability of APACHE II scores for medical-surgical intensive care patients: a prospective blinded study.. Am J Crit Care.

[16] Knaus WA, Draper EA, Wagner DP, Zimmerman JE (1985). APACHE II: a severity of disease classification system.. Crit Care Med.

[17] Garner JS, Jarvis WR, Emori TG, Horan TC, Hughes JM (1988). CDC definitions for nosocomial infections, 1988.. Am J Infect Control.

[18] Banin E, Vasil ML, Greenberg EP (2005). Iron and *Pseudomonas aeruginosa* biofilm formation.. Proc Natl Acad Sci U S A.

[19] Malhotra-Kumar S, Abrahantes JC, Sabiiti W, Lammens C, Vercauteren G (2010). Evaluation of chromogenic media for detection of methicillin-resistant *Staphylococcus aureus*.. J Clin Microbiol.

[20] Summers WC, Brookings ES, Waites KB (1998). Identification of oxacillin-susceptible and oxacillin-resistant *Staphylococcus aureus* using commercial latex agglutination tests.. Diagn Microbiol Infect Dis.

[21] Murchan S, Kaufmann ME, Deplano A, de Ryck R, Struelens M (2003). Harmonization of pulsed-field gel electrophoresis protocols for epidemiological typing of strains of methicillin-resistant *Staphylococcus aureus*: a single approach developed by consensus in 10 European laboratories and its application for tracing the spread of related strains.. J Clin Microbiol.

[22] Tenover FC, Arbeit RD, Goering RV, Mickelsen PA, Murray BE (1995). Interpreting chromosomal DNA restriction patterns produced by pulsed-field gel electrophoresis: criteria for bacterial strain typing.. J Clin Microbiol.

[23] Merlino J, Siarakas S, Robertson GJ, Funnell GR, Gottlieb T (1996). Evaluation of CHROMagar Orientation for differentiation and presumptive identification of Gram-negative bacilli and *Enterococcus* species.. J Clin Microbiol.

[24] Sarraf-Yazdi S, Sharpe M, Bennett KM, Dotson TL, Anderson DJ (2012). A 9-Year retrospective review of antibiotic cycling in a surgical intensive care unit.. J Surg Res.

[25] van Loon HJ, Vriens MR, Fluit AC, Troelstra A, van der Werken C (2005). Antibiotic rotation and development of Gram-negative antibiotic resistance.. Am J Respir Crit Care Med.

[26] Cadena J, Taboada CA, Burgess DS, Ma JZ, Lewis JS 2nd, et al (2007). Antibiotic cycling to decrease bacterial antibiotic resistance: a 5-year experience on a bone marrow transplant unit.. Bone Marrow Transplant.

[27] Sullivan A, Edlund C, Nord CE (2001). Effect of antimicrobial agents on the ecological balance of human microflora.. Lancet Infect Dis.

[28] Beardmore RE, Pena-Miller R (2010). Antibiotic cycling versus mixing: The difficulty of using mathematical models to definitively quantify their relative merits.. Math Biosci Eng.

[29] Martinez JA, Nicolas JM, Marco F, Horcajada JP, Garcia-Segarra G (2006). Comparison of antimicrobial cycling and mixing strategies in two medical intensive care units.. Crit Care Med.

[30] Jernberg C, Lofmark S, Edlund C, Jansson JK (2010). Long-term impacts of antibiotic exposure on the human intestinal microbiota.. Microbiology.

[31] Gilbert DN, Moellering RC, Elioppoulos GM, Chambers HF, Saag MS, editors (2009). Sanford Guide to Antimicrobial Therapy..

[32] Westphal JF, Brogard JM, Caro-Sampara F, Adloff M, Blickle JF (1997). Assessment of biliary excretion of piperacillin-tazobactam in humans.. Antimicrob Agents Chemother.

[33] Antonopoulos DA, Huse SM, Morrison HG, Schmidt TM, Sogin ML (2009). Reproducible community dynamics of the gastrointestinal microbiota following antibiotic perturbation.. Infect Immun.

[34] Paterson DL (2004). “Collateral damage” from cephalosporin or quinolone antibiotic therapy.. Clin Infect Dis.

[35] de Araujo OR, da Silva DC, Diegues AR, Arkader R, Cabral EA (2007). Cefepime restriction improves Gram-negative overall resistance patterns in neonatal intensive care unit.. Braz J Infect Dis.

[36] Nerandzic MM, Donskey CJ (2011). Effect of ceftobiprole treatment on growth of and toxin production by *Clostridium difficile* in cecal contents of mice.. Antimicrob Agents Chemother.

[37] Owens RC, Donskey CJ, Gaynes RP, Loo VG, Muto CA (2008). Antimicrobial-associated risk factors for *Clostridium difficile* infection.. Clin Infect Dis.

[38] Lepelletier D, Caroff N, Riochet D, Bizouarn P, Bourdeau A (2006). Role of hospital stay and antibiotic use on *Pseudomonas aeruginosa* gastrointestinal colonization in hospitalized patients.. Eur J Clin Microbiol Infect Dis.

[39] Rahal JJ, Urban C, Horn D, Freeman K, Segal-Maurer S (1998). Class restriction of cephalosporin use to control total cephalosporin resistance in nosocomial *Klebsiella*.. Jama.

[40] Borg MA, Zarb P, Scicluna EA, Rasslan O, Gur D (2010). Antibiotic consumption as a driver for resistance in *Staphylococcus aureus* and *Escherichia coli* within a developing region.. Am J Infect Control.

[41] Villers D, Espaze E, Coste-Burel M, Giauffret F, Ninin E (1998). Nosocomial *Acinetobacter baumannii* infections: microbiological and clinical epidemiology.. Ann Intern Med.

[42] Song X, Perencevich E, Campos J, Short BL, Singh N (2010). Clinical and economic impact of methicillin-resistant *Staphylococcus aureus* colonization or infection on neonates in intensive care units.. Infect Control Hosp Epidemiol.

[43] Schweizer ML, Furuno JP, Harris AD, Johnson JK, Shardell MD (2011). Comparative effectiveness of nafcillin or cefazolin versus vancomycin in methicillin-susceptible *Staphylococcus aureus* bacteremia.. BMC Infect Dis.

[44] Paul M, Yahav D, Fraser A, Leibovici L (2006). Empirical antibiotic monotherapy for febrile neutropenia: systematic review and meta-analysis of randomized controlled trials.. J Antimicrob Chemother.

[45] Towne TG, Lewis JS, Echevarria K (2009). Efficacy and safety of cefepime.. Lancet Infect Dis 9: 4–6; author reply 6–7.

[46] Leibovici L, Yahav D, Paul M (2010). Excess mortality related to cefepime.. Lancet Infect Dis.

[47] Kim PW, Wu YT, Cooper C, Rochester G, Valappil T (2010). Meta-analysis of a possible signal of increased mortality associated with cefepime use.. Clin Infect Dis.

[48] Kim PW, Nambiar S (2010). Reply to Leibovici *et. al.*. Clin Infect Dis.

[49] Leibovici L, Yahav D, Paul M (2010). Meta-analysis of a possible signal of increased mortality associated with cefepime use.. Clin Infect Dis.

