# Supplementary figures and images for: The Ecology of Antibiotic Use in the ICU: Homogeneous Prescribing of Cefepime but Not Tazocin Selects for Antibiotic Resistant Infection
Source: PLoS One. 2012 Jun 25;7(6):e38719. doi: 10.1371/journal.pone.0038719 (PMC3382621; doi:10.1371/journal.pone.0038719)

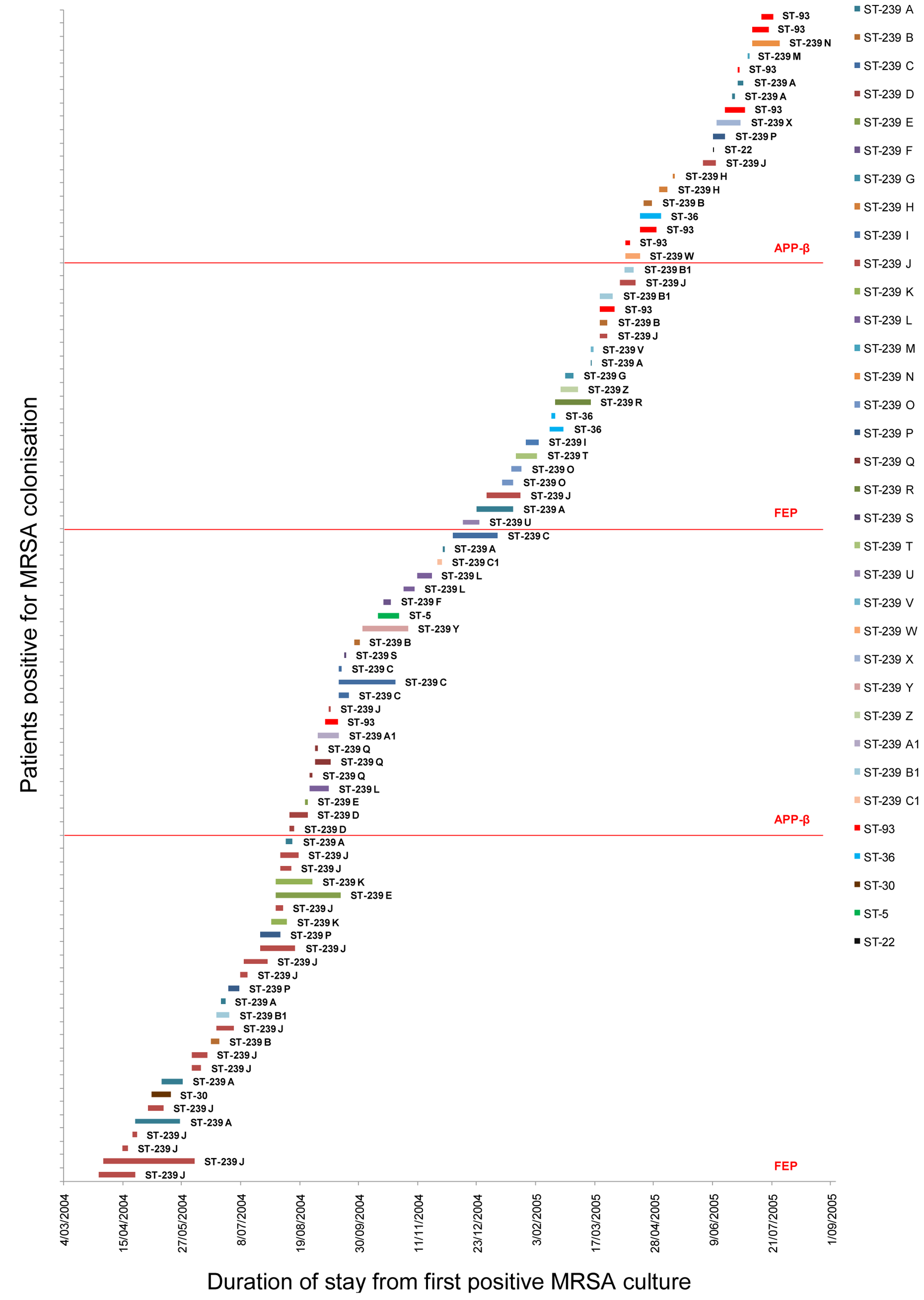

Supplement: Figure S1 — Temporal distribution of MRSA pulsotypes within cycling period. ST: Sequence type (letters indicate subgroups within STs at 95% identity). FEP: cefepime cycles; APP-β: antipseudomonal penicillin combination cycles. (TIF) [file pone.0038719.s001.tif]

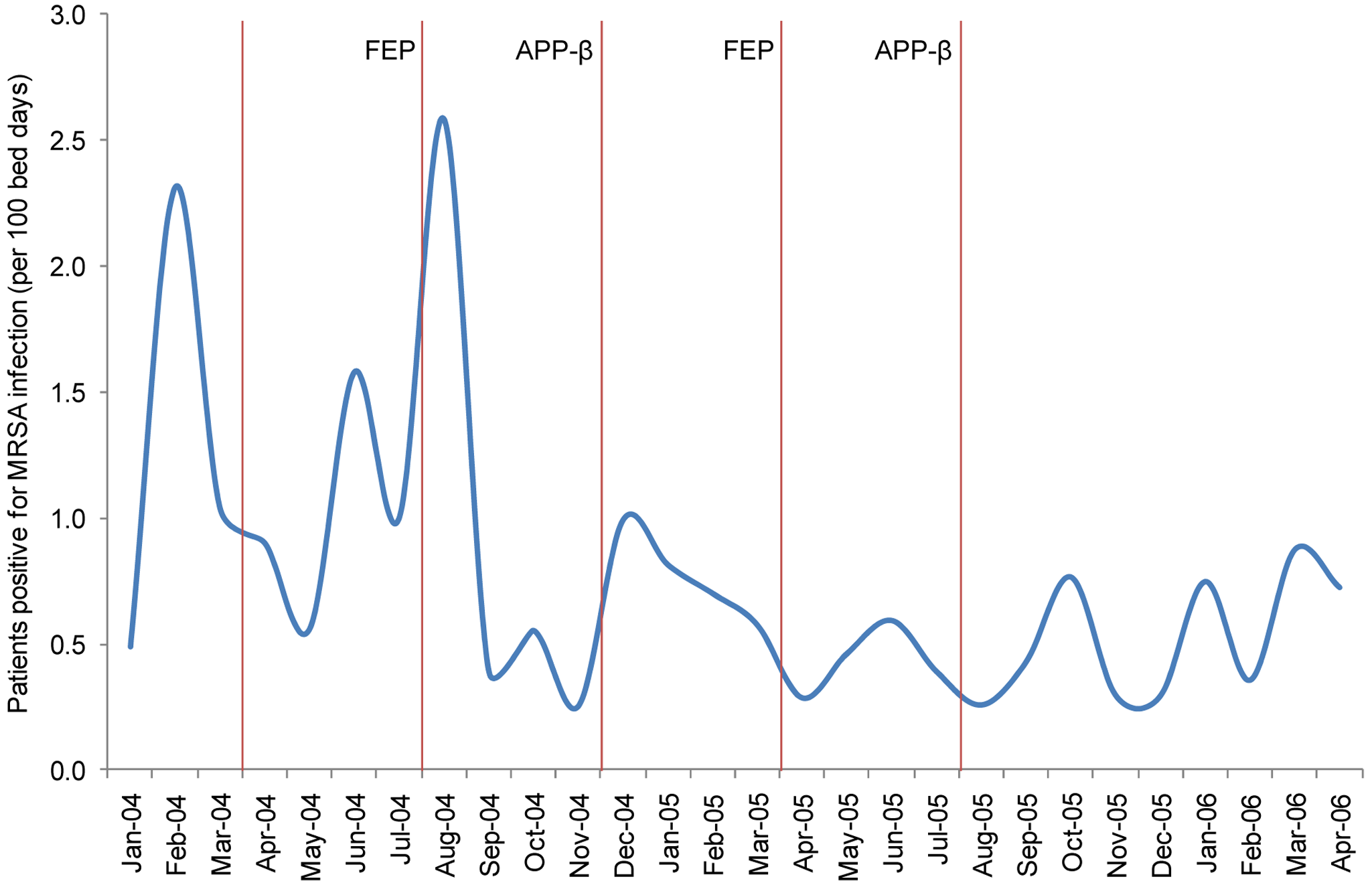

Supplement: Figure S2 — Monthly MRSA acquisition rates in Unit 1 before, during and after cycling (data from NSW Health Dept), shown as patients positive per 100 bed days. FEP: cefepime cycles; APP-β: antipseudomonal penicillin combination cycle. (TIF) [file pone.0038719.s002.tif]
