# Supplementary material for: The Ecology of Antibiotic Use in the ICU: Homogeneous Prescribing of Cefepime but Not Tazocin Selects for Antibiotic Resistant Infection
Source: PLoS One. 2012 Jun 25;7(6):e38719. doi: 10.1371/journal.pone.0038719 (PMC3382621; doi:10.1371/journal.pone.0038719)
Supplement: Table S1 — Patient characteristics of all admissions in which sampling was complete (n = 206). aMann-Whitney U test or Chi-Squared analysis. (DOC) [file pone.0038719.s003.doc]

**Table S1**. Characteristics of patients sampled before and after cycle specific treatment

| Characteristic | | Cycle 1 (n=27) | Cycle 2 (n=60) | Cycle 3 (n=65) | Cycle 4 (n=54) | p^a^ |
| --- | --- | --- | --- | --- | --- | --- |
| Age (yrs); Median (IQR) / Mean ± SD | | 62·0 (53·0 - 72·0) / 60·41 ± 15·32 | 56·0 (38·5 - 70·0) / 53·42 ± 19·79 | 52·0 (39·0 - 70·0) / 53·14 ± 20·02 | 45·0 (24·0 - 63·0) / 46·35 ± 21·59 | 0·024 |
| Male, n (%) | | 17 (63·0) | 46 (76·7) | 42 (64·6) | 40 (74·1) | 0·356 |
| ICU LOS (days); Median (IQR) / Mean ± SD | | 13·7 (10·2 - 20·9) / 16·31 ± 9·73 | 9·1 (6·2 - 14·9) / 12·39 ± 10·74 | 9·0 (6·5 - 14·4) / 14·48 ± 19·41 | 12·6 (8·7 - 16·0) / 14·02 ± 8·14 | 0·633 |
| Hospital LOS (days); Median (IQR) / Mean ± SD | | 28·3 (22·0 - 65·0) / 43·26 ± 28·67 | 27·2 (13·7 - 40·6) / 32·22 ± 23·41 | 29·0 (16·2 - 51·9) / 50·48 ± 72·66 | 34·6 (19·1 - 56·5) / 45·11 ± 44·79 | 0·225 |
| APACHE II; Median (IQR) / Mean ± SD | | 20·0 (17·5 - 28·0) / 21·96 ± 7·51 | 22·0 (17·0 - 28·0) / 22·33 ± 7·22 | 20·0 (16·0 - 25·0) / 20·35 ± 6·88 | 18·0 (14·0 - 21·0) / 20·76 ± 6·98 | 0·057 |
| Admission category: | Surgical, n (%) | 10 (37·0) | 13 (21·7) | 19 (29·2) | 20 (37·0) | 0·272 |
|  | Trauma, n (%) | 5 (18·5) | 19 (31·7) | 25 (38·5) | 21 (38·9) | 0·242 |
|  | Medical, n (%) | 10 (37·0) | 28 (46·7) | 20 (30·8) | 13 (24·1) | 0·072 |
| Operative Intervention, n (%) | | 11 (40·7) | 25 (41·7) | 34 (52·3) | 34 (63·0) | 0·096 |
| Intercranial drain or monitor, n (%) | | 7 (25·9) | 8 (13·3) | 13 (20·0) | 17 (31·5) | 0·118 |
| Intercostal Drain, n (%) | | 3 (11·1) | 6 (10·0) | 6 (9·2) | 5 (9·3) | 0·992 |
| Nasogastric catheter, n (%) | | 26 (96·3) | 49 (81·7) | 59 (90·8) | 54 (100·0) | 0·005 |
| Endotracheal Tube or Tracheostomy, n (%) | | 26 (86·3) | 53 (88·3) | 59 (90·8) | 52 (56·3) | 0·343 |
| Urinary catheter, n (%) | | 26 (96·3) | 55 (91·7) | 61 (93·8) | 54 (100·0) | 0·200 |
| Arterial catheter, n (%) | | 26 (96·3) | 53 (88·3) | 61 (93·8) | 54 (100·0) | 0·063 |
| Central venous catheter, n (%) | | 26 (96·3) | 50 (83·3) | 55 (84·6) | 54 (100·0) | 0·007 |
| Other vascular (dialytic) catheter, n (%) | | 2 (7·4) | 6 (10·0) | 9 (13·8) | 2 (3·7) | 0·287 |
| Dialysis, n (%) | | 0 (0·0) | 2 (3·3) | 8 (12·3) | 1 (1·9) | 0·023 |
| ICU Mortality, n (%) | | 2 (7·4) | 4 (6·7) | 3 (4·6) | 4 (7·4) | 0·920 |
| Hospital Mortality, n (%) | | 2 (7·4) | 5 (8·3) | 4 (6·2) | 5 (9·3) | 0·933 |

**Table S1**. Patient characteristics of all admissions in which sampling was complete (n=206). ^a^Mann-Whitney U test or Chi-Squared analysis
